# An "Unvarnished Tale."

**Published:** 1888-09-15

**Authors:** 


### The Hospital

A Weekly Institutional Journal of

**Science, Medicine, Nursing, and Philanthropy.**

*For Hospitals, Asylums, Medical Practitioners, Students, Nurses, Families, Parishes, Congregations, and the Charitable Public.*

Vol. IV. No. 103.] [September 15, 1888.

### An "Unyarnished Tale."

Many young men, by consent or desire of their parents and guardians, are presenting themselves for the first time before the peculiar custodians who guard the entrance to the medical profession. In olden times, when adventurous youths started out in search of fortune, their way was not seldom barred by glittering steel and coats of mail. In these days the soldier is replaced by the examiner, who sometimes proves a much more formidable person. Many youths who decide upon becoming doctors have not the least idea of what lies before them, and their responsible advisers are often as ignorant as themselves. It is a very common piece of advice to "look before you leap." A large proportion of those who become medical students "leap" first and "look" afterwards. Not a few soon wish they had looked first, for in that case they would never have leaped at all.

All sensible parents who have sons whom they design for doctors, and all sons who are so designated, will be glad of some little information about the region which they propose to invade. The word "invade" is not used without consideration. The medical profession, like the professions of law and divinity, is more or less of the nature of a closed preserve, which cannot be entered without difficulty. The first consideration, therefore, which should engage the attention of all who are directly concerned, is the value of the game which the covert contains. Those who are outside think they know the value of it. Those who are inside are sure they know it. We have no hesitation in agreeing with the insiders, that whether they know the value perfectly or not, they at least know it better than those who have had no personal experience at all. Even the outsiders themselves will probably concede so much.

The medical profession has undoubtedly a definite and appreciable value. The education which is compulsory before a degree can be achieved is of the highest possible worth. It is an education in things, not in words, in tangible realities, not in mere speculations. A medical student learns very early a good deal about the material of which the world is made. From the matter of which the earth consists he passes on to the vegetable and animal forms which people it. Here is a whole world of wonders opened up before him, and opened more clearly and extensively in the medical school than elsewhere. From form to function is but a step; from regular to aberrant function is another. The sight of aberrant structure and function arouses the desire to restore the normal condition; in other words, to put right that which nature permits and sometimes even encourages to go wrong. This is the beginning of the art of healing. Every step taken to acquire the preliminary knowledge of chemistry, anatomy, physiology, and pathology, on which that art is based, is of the profoundest interest and importance to the mind of broad and comprehensive powers. We affirm deliberately that every cultured man would be incalculably advantaged by a scientific training in this measure of medical study; nay, more, we go so far as to say that every man must regard his culture as incomplete and disabling who has not acquired at least a thoroughly intelligent elementary knowledge of these subjects. To make a "full man," as Bacon expresses it, a broad and comprehensive knowledge of physical science is absolutely indispensable. From the standpoint of intellectual culture, medical education is of a value which cannot be exaggerated.

But how many parents elect medicine for their sons because of its advantage as a means of culture? And how many young men enter upon medical study for any such reason? Not one in a hundred. The medical profession is chosen because it is a gentlemanly calling, in which it is easier for a man of moderate ability to earn a living than at the Bar, and which does not require any special moral and religious fitness, as the Church does. In these days every young fellow wants to be a gentleman—in other words, he wants to feel himself the equal and fit associate of cultured men. That is a not ignoble ambition. Few young men of spirit can consign themselves without a regret to a way of life which will shut them out of the society of men of education.

It is possible, however, to buy gold too dear. The life is more than meat; food, clothing, and house-accommodation are more important than frock-coats and association with men of academic standing. Common sense still has its value, and if it is despised, necessity is a task-mistress who knows how to punish. She still has her fool's cap for the blockhead, and her birch for the unmanageable. To be a gentleman a man must first of all have at least the means of livelihood. It is also desirable that he have the means for a moderate degree of simple refinement in daily life. Will the medical profession ensure bread and butter to those who follow it? That is the initial interrogation: it is the question of questions. The answer is stern and uncompromising: It will not ensure bread and butter, or even bread alone, if men continue to enter it as rapidly as they do now. Nay, more, it does not by any means ensure bread without butter to all its deserving members, even at the present time.

A few plain facts are worthy of sober consideration. The University of Edinburgh is the largest and most important medical school in the United Kingdom.

Thirty years ago its medical students numbered about four hundred, to-day they number eighteen hundred. They are more than four times as numerous as they were in 1858. This is not a solitary instance, although it is so striking. The medical schools in London, Manchester, Birmingham, Leeds, Liverpool, Oxford, Cambridge, and Durham, are numbered by the dozen. Scotland alone counts six or seven, and Ireland about the same number. Every colony of any importance has its hospital, and its physicians, surgeons, and students. Almost all these numerous schools of medical study have increased in a ratio approaching that of the University of Edinburgh during the same period. The population of the empire, on the other hand, has not grown with half the rapidity. The natural result is that there are now, in almost every county, two medical men where there used to be only one, whilst in many large cities, such as London, the proportion is as three to one Besides, the modern doctor is of an entirely different order from his predecessor of thirty years ago. His culture is that of a man of education and a gentleman; his wants and his power of spending money have correspondingly increased. The pressure and competition between medical men at the present time is keen beyond description. The fee of the average practitioner in many places has not got beyond the half crown which was the standard fee half a century ago, whilst in vast numbers of instances, as is well known, it has gone down to two shillings, one shilling, and even sixpence. The curate and the poor incumbent are the pain and scandal of modern times; the poor medical man is rapidly falling to the same terrible level. This is no highly-coloured picture, but a plain statement of facts. Slavery and semi-starvation are the lot of vast numbers of deserving medical practitioners.

Such being the country which lies open to ambition, the question is: What kind of soldiers should they be who make up their minds to invade it? The stupid had better stay at home; the impatient will be well advised to choose some other calling; the very poor start at a great disadvantage, and the disadvantage clings to them throughout the whole of life; the idle and the dissipated are entirely out of place, both in the medical school and in the ranks of the profession. They never had much chance of success; now they have none at all. All who cannot both work and wait, who cannot practice both perseverance and self-denial, may make up their minds to failure, mortification, and a life of trouble. This is inevitable, and cannot be otherwise. On the other hand, to the man of steadfast work, sound common sense, and good feeling, medicine offers a field worthy of his highest and noblest ambition. It is an absolutely honest and also a most useful calling. To those whose merit is undoubted, it offers a modest income, a fair worldly position, and the respect and gratitude of the better portion of their fellow-men. The prizes of the Bar and of the Church have no equals in medicine But there is for the doctor of sterling worth a place and a consideration in the hearts of men and women which Archbishops might envy, and to which Lord Chancellors never attain.

